# Effects of Casein Hydrolysate Ingestion on Thermoregulatory Responses in Healthy Adults during Exercise in Heated Conditions: A Randomized Crossover Trial

**DOI:** 10.3390/nu12030867

**Published:** 2020-03-24

**Authors:** Yasuyuki Sakata, Chikako Yoshida, Yuka Fujiki, Yutaka Matsunaga, Hirohiko Nakamura, Takashi Shimizu, Yasuhiro Takeda, Tatsuro Amano

**Affiliations:** 1 Wellness & Nutrition Science Institute, Morinaga Milk Industry Co., Ltd., Zama, Kanagawa-Pref. 252-8583, Japan; ys-sakata@morinagamilk.co.jp (Y.S.); y_matsunaga@idaten.c.u-tokyo.ac.jp (Y.M.); hi_nakam@morinagamilk.co.jp (H.N.); t_simizu@morinagamilk.co.jp (T.S.); ya_taked@morinagamilk.co.jp (Y.T.); 2 Laboratory for Exercise and Environmental Physiology, Faculty of Education, Niigata University, Niigata, Niigata-Pref. 950-2181, Japan; chariiiiiin@gmail.com (C.Y.); yuka.fujiki28@gmail.com (Y.F.)

**Keywords:** casein hydrolysate, sweat rate, body temperature, thermoregulation, exhaustion, aerobic exercise, heat

## Abstract

Food ingestion has been shown to affect thermoregulation during exercise, while the impact of protein degradant consumption remains unclear. We investigated the effects of casein hydrolysate ingestion on thermoregulatory responses during exercise in the heat. In a randomized, placebo-controlled, double-blind, crossover trial, five men and five women consumed either 5 g of casein hydrolysate or placebo. Thirty minutes after ingestion, participants cycled at 60% VO_2_max until voluntary exhaustion wearing a hot-water (43 °C) circulation suit. Exercise time to exhaustion, body core temperature, forearm sweat rate, and forearm cutaneous vascular conductance did not differ different between the conditions. However, chest sweat rate and mean skin temperature increased upon casein hydrolysate ingestion compared with placebo during exercise. Increased chest sweat rate upon casein hydrolysate ingestion was associated with elevated sudomotor sensitivity to increasing body core temperature, but not the temperature threshold for initiating sweating. A positive correlation was found between chest sweat rate and plasma total amino acid concentration during exercise. These results suggest that casein hydrolysate ingestion enhances sweating heterogeneously by increasing peripheral sensitivity of the chest’s sweating mechanism and elevating skin temperature during exercise in the heat. However, the physiological link between plasma amino acid concentration and sweat rate remains unclear.

## 1. Introduction

Heat stress attenuates aerobic exercise performance due to impairment of integrative physiological mechanisms [1,2] including thermoregulatory impairments that result in hyperthermia [3,4] and/or high skin temperature [5,6] during exercise. Several factors potentially affect core body temperature (Tco) and skin temperature independent of exercise-induced heat production. For example, food ingestion has been shown to elevate Tco associated with an increase in postprandial energy expenditure [7,8] which delays Tco threshold for thermoregulatory cutaneous vasodilation during exercise [9]. The type of nutrients also influences postprandial energy expenditure and possibly Tco since a greater energy consumption has been reported after protein intake compared with an equivalent amount of carbohydrate ingestion through encouraging protein synthesis and mitochondrial enzymatic activity [10,11]. Differences in protein source may also affect postprandial energy expenditure through the differences in amino acid compositions and its absorption kinetics [12]. Studies have reported that peptides included in casein hydrolysate (CH) show faster absorption kinetics relative to those of amino acids [13]. This is also evidenced by the fact that the ingestion of CH induces a greater energy expenditure, probably due to enhanced protein synthase and a promotion of mitochondrial enzyme activity than those of equivalent composition of amino acids [14,15]. Overall these previous studies imply that proteins consumed before exercise, especially protein hydrolysate such as CH, may influence thermoregulatory responses during exercise. However, to the best of our knowledge, there is limited available literature in this area of research.

Oral consumption of branched-chain amino acids (BCAA), tyrosine, or arginine prior to exercise do not influence Tco, skin temperature, skin blood flow, and sweat rate estimated from exercise-induced body mass loss as well as endurance exercise performance in the heat [16,17,18]. These previous studies suggest that amino acid consumption does not alter thermoregulatory responses and exercise performance in the heat. On the other hand, intravenous infusion of a mixture of 20 amino acids, which are found in proteins, increased Tco and delayed Tco thresholds for sweating and cutaneous vasodilation in resting heated humans [19], indicating a suppressive modulation for physiological heat loss regulation [20]. The precise reasons for the observed discrepancies between these previous studies regarding the effects of amino acid ingestion/infusion on human thermoregulation are unknown. However, they might be related to the differences in the methodology employed (e.g., exercise vs. passive heating, oral consumption vs. intravenous infusion, and whole body sweat loss vs. continuous local sweat rate measurement). Nevertheless, these observations cannot be translated into the potential effects of CH ingestion since CH could elevate Tco more than of amino acid ingestion because of potentially greater postprandial energy expenditure in CH ingestion [14]. Consequently, the influence of CH ingestion on thermoregulatory responses in the heat requires further research.

The present study investigated the effect of CH ingestion on thermoregulatory responses during exercise in the heat (exercise wearing a hot-water circulating perfusion garment). In a preliminary test, we investigated plasma amino acid concentrations after CH ingestion under a resting normothermic condition wearing a 34 °C water circulating suit to determine the timing of CH ingestion before exercise. In the main experiment, we tested a hypothesis that CH ingestion before exercise would elevate Tco and suppress heat loss responses evidenced by an elevation of Tco threshold for initiating heat loss during exercise. 

## 2. Materials and Methods

### 2.1. Ethical Approval

The present study was approved by the human ethical committee of Niigata University, Niigata, Japan (2017-3-008) and conformed to the standards set by the latest version of the Declaration of Helsinki. The study protocol was registered at the University Hospital Medical Information Network (UMIN) clinical trial registry (ID: UMIN000030145). All participants provided verbal and written informed consent before participating in the study.

### 2.2. Study Design

To determine the timing of CH ingestion prior to the exercise, we measured plasma amino acid concentration after CH ingestion under resting normothermic conditions (preliminary test). In this preliminary test, we also evaluated thermoregulatory variables to explore the potential influence of CH intake at rest. Then, we investigated the effect of CH ingestion on thermoregulatory responses and exercise tolerance under heated conditions (main experiment). All experiments were performed in a double-blind, placebo-controlled, randomized, crossover manner. Randomization was performed by the permuted block method (block size of four) and was stratified by gender using SAS ver. 9.4 (SAS Institute Inc., Cary, NC, USA). All trials were conducted between November 2017 and December 2017 at Niigata University, Niigata, Japan.

### 2.3. Participants

Ten healthy adults (five men and five women) participated in the preliminary and main experiments, respectively. Age, height, and body weight were 21.4 ± 0.8 years, 167.7 ± 3.7 cm, and 59.8 ± 7.4 kg in the preliminary test and 21.3 ± 0.8 years, 166.7 ± 4.4 cm, and 62.0 ± 4.7 kg in the main experiment, respectively. Maximum oxygen uptake (VO_2_max), which was measured only in the main experiment, was 35.2 ± 8.2 mL/kg/min. Individuals with food allergies, who were smokers, habitually consumed protein and/or amino acid supplements, and were on any medication or oral contraceptives (only females) were excluded from the study. Due to the limited study duration, we did not take into account the menstrual cycle of female participants.

### 2.4. Supplementation

The test food contained 6.1 g CH (21 kcal; protein, 5.0 g; carbohydrate, 0.3 g), whereas the placebo (PL) contained 6.1 g indigestible dextrin (7 kcal; protein, 0 g; carbohydrate, 5.7 g). Caramel coloring agent, sweetener, and coffee flavoring agent were included in both types of food to prevent identification of the foods by taste, flavor, or color. Test food and PL were dissolved in 200 mL non-caffeine barley tea prior to ingestion. The amino acid composition of CH, which is shown in Table 1, was measured by an amino acid analyzer after hydrolyzation with hydrogen chloride or barium hydroxide as previously reported [21]. The amount of supplementation was decided based on a previous study describing CH ingestion in humans [22].

### 2.5. Experimental Protocol

#### 2.5.1. Preliminary Test

Experiments were conducted in an open-space laboratory under thermoneutral conditions (~25 °C and ~50% relative humidity). Participants reported to the laboratory separated by a minimum of seven days after consumption of the test food or PL. Participants were instructed to refrain from consuming alcohol or caffeine, and from participating in any strenuous physical activity at least 24 hours prior to each experimental trial. Additionally, the night before the experimental session, they consumed a standardized meal (energy content, ~750 kcal) consisting of ~115 g of carbohydrate, ~20 g of fats, and ~27 g of protein. The meal included 500 mL of non-caffeinated Japanese tea. In addition, participants were asked to consume 500 mL of water before going to bed the night prior to each experiment.

**Figure 1:**
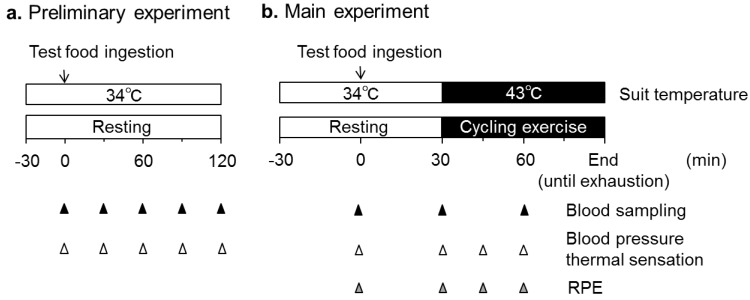
Schematic timeline of the experimental protocols for the preliminary and main experiments. RPE, rating of perceived exhaustion.

Participants reported to the laboratory between 7:00 and 9:00 am in a fasted state except for 500 mL water at least one hour before their arrival. Upon the arrival of participants, urine samples were collected to measure urine specific gravity (USG) and assess hydration status [23]. Then, the participants’ body mass and height were measured using a platform balance (HW-100KC; A & D, Tokyo, Japan) and a height meter (YS501-P; Sanyu, Tokyo, Japan), respectively. Thereafter, the participants donned a water perfusion suit (Allen-Vanguard, Ottawa, Canada) over shorts and a cotton T-shirt, which covered their whole bodies except for their hands, feet, and head. Skin temperature is known to differ between individuals [24,25,26] and could influence sweating and cutaneous vascular responses independent of other modulators [20,27]. We therefore used the water perfusion suit to clamp skin temperature to minimize inter- and intra-individual variations in this variable. The temperature of the water circulating the suit was maintained as similar to that of skin temperature in normothermic humans at 34 °C throughout the experiment to mimic a normothermic state [28,29,30,31]. The instrumentation was approximately 60 min. After that, baseline (BL) data were collected for 5 min, during which a blood sample was collected from a warmed fingertip. Subsequently, participants ingested the test food or PL dissolved in barley tea as described above and rested for 2 h in a semi-supine position (Figure 1a). Ingestion of the test food or PL during the first experiment day was randomly assigned, and the washout period was at least seven days.

#### 2.5.2. Main Experiment 

General preparations before the food ingestion were the same as the preliminary test, while VO_2_max was assessed at least one week before the main experiment. After consuming the test drink, participants remained in a resting position for 30 min, which was determined from the preliminary test to maximize the plasma amino acid concentrations (see results). During this 30 min, the temperature of the water circulating the suit was maintained at 34 °C. Subsequently, whole body heating was initiated by raising the temperature of the circulating water to 43 °C. At the same time, participants started cycling at 60% VO_2_max and 60 rpm. Cycling was continued until voluntary exhaustion, which was defined as the time point when the participants could no longer maintain the cadence of 50 rpm. No fluid consumption was allowed during exercise (Figure 1b).

### 2.6. Measurements

Rectal temperature as an index of Tco was measured continuously by a calibrated thermistor probe (401J; Nikkiso-thermo, Tokyo, Japan) inserted 12 cm past the anal sphincter. Skin temperature was measured continuously using a type-T thermocouple temperature probe (Inui Engineering, Higashi Osaka, Japan). Weighted mean skin temperature (Tsk) was calculated using four skin temperatures weighting according to the following regional proportions: forearm 30%, chest 30%, thigh 20%, and lower limb 20% [32]. Mean body temperature was calculated from Tco and Tsk weighting, according to the following proportions: Tco 80% and Tsk 20% [33].

Local sweat rate was measured continuously using the ventilated capsule method. A 5.3 cm^2^ plastic capsule was affixed to the forearm and chest using a topical glue (Collodion; Kanto chemical, Tokyo, Japan). The forearm was placed on a stull at a height level with the heart throughout the experiment. Dry nitrogen gas was passed through each capsule over the skin at a rate of 1.0 L/min. Water content in the effluent air was measured using a capacitance hygrometer (HMP60; Vaisala, Helsinki, Finland). Local skin blood flow on the forearm was measured continuously using laser Doppler flowmetry (FLO-C1; Omegawave, Inc, Tokyo, Japan) located adjacent to the ventilated capsule. Cutaneous vascular conductance (CVC) was calculated from the ratio of skin blood flow to mean arterial blood pressure. Systolic and diastolic blood pressures were measured every 30 (preliminary experiment) or 15 min (main experiment) on the arm contralateral to the skin blood flow and sweat rate measurements using the standard auscultatory method. The following equation was used to calculate mean arterial blood pressure: (systolic blood pressure − diastolic blood pressure)/3 + diastolic blood pressure. Heart rate was recorded using a Polar coded WearLink and transmitter and RS800 interface (Polar Electro Oy, Finland). Skin temperatures, Tco, sweat rate, and skin blood flow were recorded by a datalogger at 1-s intervals.

Blood samples were collected from a warmed fingertip prior to supplementation (BL) and every 30 min afterward in both the preliminary and main experiments. All blood samples were collected in a semi-recumbent position throughout the experiments. Collected blood samples were centrifuged and the extracted plasma samples were immediately frozen at −30 °C until the measurement of amino acids. Plasma samples were deproteinized by adding trichloroacetic acid and centrifuged at 15,000× *g* for 15 min at 4 °C; the supernatant was analyzed using an amino acid analyzer (L-8900; Hitachi High-technologies Corporation, Tokyo, Japan). Thermal sensation and rating of perceived exhaustion (RPE, main experiment only) were recorded prior to the supplementation and every 30 min (preliminary experiment) or 15 min (main experiment) afterward.

### 2.7. Data and Statistical Analyses

All continuously recorded variables were averaged over 5 min. Given that mean arterial blood pressure was recorded every 30 min (preliminary experiment) or 15 min (main experiment) during each experiment, CVC was also analyzed at the same time points. Changes in the primary outcomes (i.e., Tco, Tsk, and sweat rate) from their BL values (Δ) were calculated during exercise in the main experiment. In addition, Tco and mean body temperature thresholds and slopes for sweating at each skin site were calculated by using a segmented regression analysis method in the main experiment [34]. This analysis was not conducted for CVC due to the limited number of measurements. To explore the potential relationship between sweat production and changes in plasma amino acid concentration during exercise (main experiment), we plotted Δsweat rate against the change in plasma total amino acid (ΔTAA) concentration 60 min after the supplementation (and thus 30 min of exercise). We selected this time point to explore the relationship between Δsweat rate and ΔTAA during exercise since blood collection was only possible at this time point in most participants.

Two-way repeated measures analysis of variance (ANOVA) was used as the repeated factors of two stage-protocol (BL and every 5 min after the supplementation till the end of experiment for the preliminary experiment and till 55 min (25 min of exercise) in the case of the shortest exercise duration observed for the main experiment) and the supplementations (CH and PL) for variables measured in both the preliminary and main experiments. Plasma TAA concentration, mean arterial blood pressure, CVC, thermal sensation, and RPE were analyzed by using two-way repeated measures ANOVA as the repeated factors of the protocol stage (BL and every 15 or 30 min after the supplementations) and test food as well as PL. Huynh–Feldt correction was applied under violation of the assumption of sphericity. Post hoc analyses were performed using a Bonferroni corrected paired *t*-test. Thresholds of Tco and mean body temperature, and slopes for sweating and TTE were compared between the conditions with a paired *t*-test. The relationship between Δsweat rate and ΔTAA was evaluated using the Pearson’s correlation test. Due to technical difficulties, some variables were only analyzed in a limited number of participants, and indicated in the figure legends and tables. All data are expressed as mean ± SD. Segmented linear analysis was performed by using GraphPad Prism version 8.3.0 (GraphPad Software, La Jolla, CA). All statistical analyses were performed by using SPSS version 22.0 (IBM, Armonk, NY).

## 3. Results

### 3.1. Preliminary Experiment

**Figure 2:**
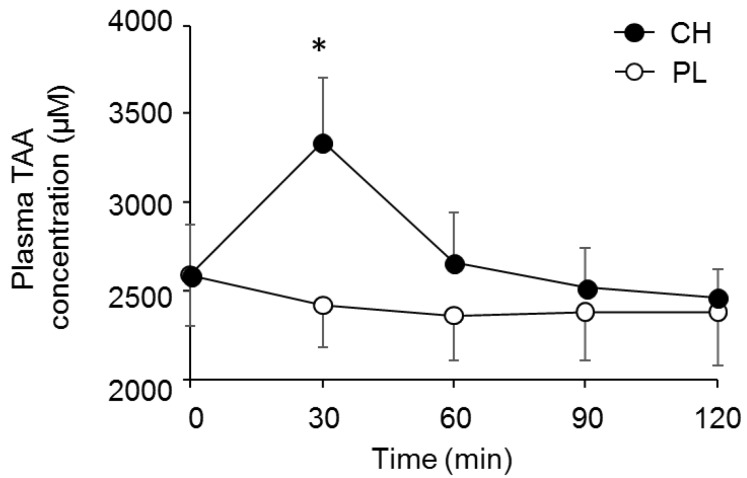
Plot showing the trend of plasma total amino acid (TAA) concentration as a function of time following casein hydrolysate (CH) or placebo (PL) ingestion (*n* = 9) in the preliminary test. Values are expressed as mean ± SD. *, *P* < 0.001.

A supplementation x time interaction effect was observed for plasma TAA (*P* < 0.001, Figure 2). Post hoc analysis revealed that plasma TAA concentration in CH was significantly higher compared with that in PL at 30 min after ingestion (*P* < 0.001), although it later returned to a similar level to that in PL (Figure 2). There were no differences in other variables between CH and PL conditions throughout the experiment (all variables showed *P* > 0.05, Table 2). Measurable sweat rate was not observed throughout the preliminary experiment in both CH and PL trials (data not shown).

### 3.2. Main Experiment

**Figure 3:**
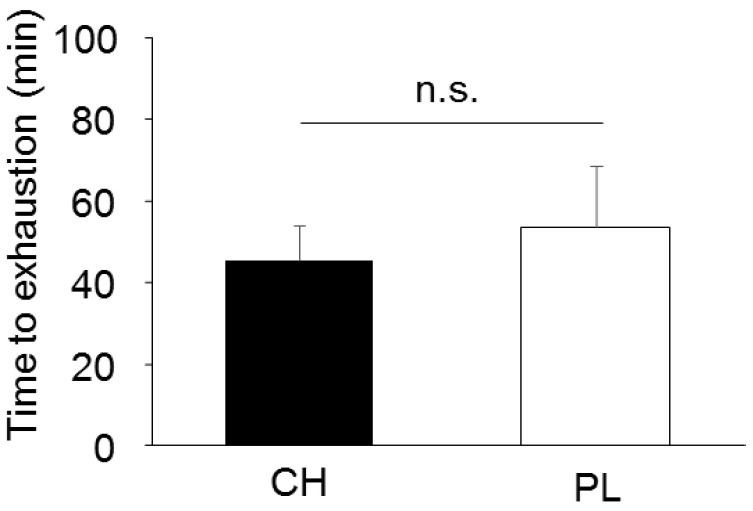
Time to exhaustion in casein hydrolysate (CH) and placebo (PL) conditions during exercise in the main experiment (*n* = 10). Values are expressed as mean ± SD.

**Figure 4:**
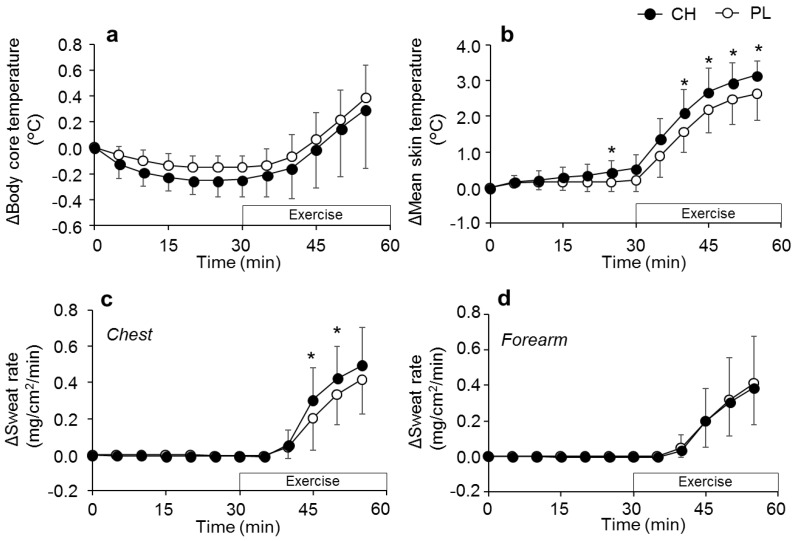
Changes in (**a**) body core temperature (*n* = 9), (**b**) mean skin temperature (*n* = 8), (**c**) chest sweat rate (*n* = 9), and (**d**) forearm sweat rate (*n* = 10) following casein hydrolysate (CH) or placebo (PL) ingestions in the main experiment. Values are expressed as mean ± SD. *, *P* < 0.05.

No difference in TTE during exercise was observed between the conditions (Figure 3). Throughout the experiment, Tco and ΔTco were also not different between the conditions (both *P* > 0.05, Figure 4a and Table 3). Significant supplementation x time effect was observed in ΔTsk (*P* = 0.035), and post hoc analysis revealed higher ΔTsk in CH compared with that in PL at 25 min and from 40 min to 55 min after supplementation (Figure 4b). While Tsk showed significant interaction effect between the conditions (*P* = 0.035), post hoc analysis revealed no difference in this parameter at any time point between PL and CH (Table 3). Significant supplementation x time effect was observed in the Δsweat rate on the chest (*P* = 0.023), which indicated a higher Δsweat rate in CH compared with PL at 45 and 50 min after supplementation (*P* < 0.05 for both time points, Figure 4c). On the other hand, Δsweat rate on the forearm was not significantly different between the conditions (*P* = 0.805 for interaction, Figure 4d). No differences in body mass loss were observed between supplementations during the experiment (0.62 ± 0.15 and 0.77 ± 0.40% for CH and PL, respectively). The end exercise Tco, heart rate, and Tsk were 37.54 ± 0.31 and 37.55 ± 0.48 °C, 155.8 ± 12.0, and 161.8 ± 6.9 beats/min, and 34.96 ± 0.59 and 34.79 ±0.76 °C for CH and PL, respectively (all *P* > 0.05).

The elevated Δsweat rate on the chest in CH relative to that in PL was associated with higher slopes for sweating to increasing Tco (*P* = 0.019) and mean body temperature (*P* = 0.090, Table 4). For both chest and forearm sweat rate, Tco thresholds in CH were significantly higher than that in PL (*P* = 0.005 and *P* = 0.006, respectively), while this difference was not observed in both ΔTco and Δmean body temperature thresholds. Plasma TAA was higher in CH than that in PL at 30 min after supplementation (Table 3). 

**Figure 5:**
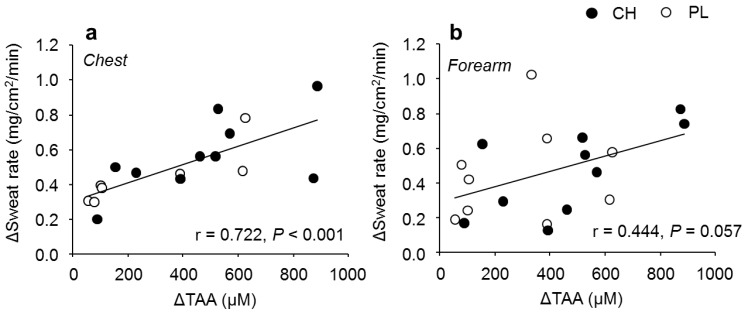
Relationship between changes in sweat rate (Δsweat rate) and plasma total amino acid (ΔTAA) concentration at 60 min after casein hydrolysate (CH) or placebo (PL) ingestion in the main experiment. Relationships (**a**) between Δsweat rate on the chest and ΔTAA (*n* = 18), and (**b**) between Δsweat rate on the forearm and ΔTAA (*n* = 19).

At 60 min after supplementation, the Δsweat rate on the chest was positively correlated with ΔTAA (*r* = 0.722, *P* < 0.001, Figure 5a), while no significant correlation was observed between Δsweat rate on the forearm and ΔTAA (*r* = 0.444, *P* = 0.057, Figure 5b). The positive correlation between Δsweat rate on the chest and ΔTAA was particularly significant in the PL group (*r* = 0.817, *P* = 0.013, *n* = 8 in PL, *r* = 0.636, *P* = 0.066, *n* = 9 in CH). There were no differences in other variables between CH and PL throughout the experiment (Table 3).

## 4. Discussion

Contrary to our hypothesis, we found that CH ingestion did not influence Tc elevation during exercise in the heat. We observed higher Tco thresholds for sweating in CH compared with that in PL. However, this response might be due to a slightly higher Tco before CH ingestion when compared with PL supplementation and not due to CH ingestion itself (see discussion below), which contradicts our hypothesis. Interestingly, CH ingestion increased Tsk and chest sweat rate without affecting responses on the forearm. Higher chest sweat rate in CH compared with that in PL was associated with greater sudomotor sensitivity to increasing Tco and possibly higher plasma amino acid concentration. We did not observe any influence of CH ingestion on TTE. These results suggest that CH ingestion prior to exercise elevates thermoregulatory sweating and skin temperature without affecting Tco during exercise in the heat.

Several studies have reported that amino acid ingestion does not affect Tco during exercise [16,17,18]. Contrary to this general observation, we originally hypothesized that CH ingestion, which increases blood amino acid concentration [35], would elevate Tco during exercise since it has been shown to increase postprandial energy expenditure to a greater extent than the equivalent amount of amino acid consumption [14]. This response, if any, might occur through diet-induced thermogenesis via mitochondrial BCAA catabolism in brown adipose tissue [11,36,37]. Despite these assumptions, we observed similar Tco between CH and PL ingestions, suggesting that CH does not affect Tco during exercise in the heat. Unexpectedly, Tco during exercise was low in both conditions. Given that the change in Tco from pre-exercise level (changes from the time point at 30 min in Figure 4a) during exercise was comparable to previous studies [16], the observed low Tco was probably due to a low resting Tco prior to the commencement of exercise. The low resting Tco at baseline was related to a female participant who showed quite a low rectal temperature, which was 34.75 and 34.66 °C for the CH and PL conditions, respectively. As we did not assess the menstrual phase in female participants in the present study, it is unclear how the menstrual phase potentially influenced the low Tco response in this participant. Thus, the precise reason(s) for the low Tco in this female participant remain unknown. Nevertheless, as we did not observe an increase in Tco after CH ingestion, it is assumed that the amount of CH consumed might be too small to affect these responses in the present study. Further studies are required to explore the impact of ingestion of a high dose of CH on human thermoregulatory responses during exercise.

We observed an elevated Tco threshold for initiating sweating upon CH ingestion. However, we consider that the observed higher Tco threshold for sweating in CH than in PL was independent of the supplementation effect because ΔTco thresholds for sweating were not different between supplementations. The resting Tco before supplementation was already slightly higher in the CH condition compared with the PL condition (Table 3), in which the response likely contributed to an elevated Tco threshold for initiating sweating during exercise, apart from the supplementation effects. Once the sweating was initiated, we observed higher chest sweat rate in the CH condition compared with the PL condition, without modulating forearm sweat rate during exercise in the heat, demonstrating heterogeneous effects of CH on sweating across skin sites. Supporting this observation, CH ingestion did not affect whole body sweating. The elevated slope for chest sweating to increasing Tco implies peripheral modification of sweating following CH ingestion [20,38,39]. Given that a similar trend was observed even with the slope calculated as a response to mean body temperature and that the local chest skin temperature was not different between supplementations (data not shown), the increased chest sweat rate following CH ingestion might be independent of skin temperature. Interestingly, we observed a positive correlation between chest sweat rate and plasma TAA concentration and a slightly weak correlation between forearm sweat rate and plasma TAA concentration at 30 min of exercise. However, the precise interpretation of this result was somewhat difficult since several studies have demonstrated that ingestion of amino acids does not affect sweat production (whole body sweating) during exercise [16,17]. In addition, it has been shown that intravenous infusion of amino acids did not alter peripheral sweat production during passive heat stress in humans [19]. Furthermore, given that we observed a co-relationship between sweat rate and TAA even in the PL condition, there is a possibility that the changes in TAA could potentially influence sweating during exercise independent of the supplementation effect per se. Thus, the relationship between amino acids and sweating is inconsistent between previous and present studies. Further studies are required to bridge the gap of knowledge between the present study and those previous studies to elucidate the impact of increased plasma amino acid concentration on sweating during exercise.

CH ingestion increased mean skin temperature compared with that of PL, however the magnitude of the elevation might be too small to affect physiological responses. We are unaware of the precise reason(s) for an increased Tsk following CH ingestion, but one can speculate that CH would elevate Tsk due to its potential heterogeneous vasodilation effect across several body sites [40,41]. However, as we measured skin blood flow only on the forearm, the trend of skin blood flow across several body sites and the precise influence of CH ingestion on skin perfusion remains unknown.

Previous studies have shown that high skin temperature (~35 °C) and dehydration (4%) attenuate endurance exercise performance in the heat [3,4,5,6]. We observed a high skin temperature where the level attenuates exercise performance [5] while the magnitude of the differences between conditions (~+0.3 °C) might not be large enough to affect the performance. Furthermore, non-uniform increases in sweating after CH ingestion implies a relatively small impact of this supplementation on whole body sweat loss and thus dehydration. We also found a high thermal sensation during exercise in both conditions, suggesting that perceptional thermal sensation might also affect exercise performance in both supplementation conditions in this study, which is in line with previously reported studies [42,43,44]. Therefore, irrespective of the differences in supplementation, we considered that the high skin temperature and thermal sensation, but not dehydration, affected exercise tolerance in the heat in this study.

There were several limitations in our study. First, we used the minimum sample size for an intervention study and thus additional experiments are required to validate our findings. Second, we did not measure resting metabolic rate after CH ingestion, which is a fundamental measurement in this type of research. Thus, future studies are required to accurately interpret the results of this study. Third, we did not control for the menstrual cycle in female participants, which might potentially affect physiological responses in this population. Fourth, we observed a slight attenuation of Tco during the resting period prior to exercise despite wearing a water perfusion suit to mimic a normothermic state. We observed a lower skin temperature (~31.6 °C) than those of comparable previous studies (~34 °C), which used a similar water perfusion suit [28,45]. We were unaware why this occurred in the present study but the low skin temperature might contribute to the attenuation in resting Tco prior to the exercise. It remained unknown if and how the slightly lowered Tco prior to the exercise affected our general findings in the present study. Finally, the usage of a water perfusion suit may limit our ability to assess the impact of CH during exercise in real-world situations such as a condition under a high ambient temperature permitting the evaporation of sweat.

## 5. Conclusions

In conclusion, this study revealed that CH ingestion prior to exercise elevated sweating by modulating peripheral sudomotor sensitivity and skin temperature without affecting Tco and exercise tolerance during exercise in the heat. The physiological link between observed amino acid concentration and sweat production during exercise needs to be explored further.

## Figures and Tables

**Table 1:** Amino acid composition of casein hydrolysate.

**Essential amino acids (mg/g protein)**					
Lys	77	Cys	3	Tyr	37
Thr	50	Ile	52	Trp	2
Val	62	Leu	81	His	25
Met	27	Phe	35		
**Non-essential amino acids (mg/g protein)**					
Asp + Asn	85	Pro	108	Arg	25
Ser	63	Gly	21		
Glu + Gln	282	Ala	34		

**Table 2:** Physiological and perceptual responses in the preliminary experiment.

		*n*	Baseline	Time after the Supplementation				Interaction
				30 min	60 min	90 min	120 min	
Heart rate(beats/min)	CH	10	72.2 ± 8.0	72.0 ± 6.7	74.8 ± 6.6	74.5 ± 7.8	76.0 ± 7.2	n.s.
	PL		71.2 ± 7.1	71.1 ± 7.5	73.0 ± 7.9	73.3 ± 8.0	74.5 ± 8.6	
Mean arterial blood pressure (mmHg)	CH	10	87.1 ± 6.1	85.2 ± 5.5	85.3 ± 5.0	84.7 ± 6.3	85.1 ± 6.8	n.s.
	PL		85.1 ± 6.8	85.2 ± 7.8	85.4 ± 7.2	86.7 ± 5.9	85.8 ± 6.1	
Body core temperature (°C)	CH	10	36.31 ± 1.01	36.24 ± 0.87	36.40 ± 0.76	36.53 ± 0.65	36.61 ± 0.56	n.s.
	PL		36.65 ± 0.43	36.52 ± 0.37	36.58 ± 0.34	36.64 ± 0.30	36.73 ± 0.28	
Mean skin temperature (°C)	CH	8	31.66 ± 0.94	31.92 ± 0.87	32.02 ± 0.91	32.13 ± 0.87	32.26 ± 0.72	n.s.
	PL		31.62 ± 0.72	31.90 ± 0.37	31.96 ± 0.42	31.91 ± 0.37	31.95 ± 0.48	
Mean body temperature (°C)	CH	8	35.29 ± 0.89	35.31 ± 0.72	35.48 ± 0.64	35.61 ± 0.55	35.72 ± 0.45	n.s.
	PL		35.62 ± 0.46	35.61 ± 0.36	35.68 ± 0.34	35.73 ± 0.30	35.81 ± 0.26	
Skin Blood Flow (AU)	CH	9	0.102 ± 0.023	0.104 ± 0.026	0.101 ± 0.022	0.101 ± 0.030	0.114 ± 0.032	n.s.
	PL		0.109 ± 0.031	0.101 ± 0.021	0.100 ± 0.020	0.102 ± 0.024	0.105 ± 0.026	
CVC (AU/mmHg)	CH	9	0.0012 ± 0.0003	0.0012 ± 0.0003	0.0012 ± 0.0003	0.0012 ± 0.0003	0.0013 ± 0.0003	n.s.
	PL		0.0013 ± 0.0004	0.0011 ± 0.0002	0.0012 ± 0.0002	0.0012 ± 0.0002	0.0013 ± 0.0003	
Thermal sensation (AU)	CH	10	3.2 ± 0.6	3.0 ± 0.8	3.0 ± 0.8	3.3 ± 0.9	3.6 ± 0.5	n.s.
	PL		3.2 ± 0.9	3.0 ± 1.1	3.2 ± 0.6	3.1 ± 0.9	3.1 ± 0.7	

Values are expressed as mean ± SD. CH, casein hydrolysate; PL, placebo; CVC, cutaneous vascular conductance; n.s., not significant.

**Table 3:** Physiological and perceptual responses in the main experiment.

		*n*	Baseline	Time after the Supplementation (Duration of Exercise)				Interaction
				15 min (−15 min)	30 min (0 min)	45 min (15 min)	55 min (25 min)	
Heart rate (beats/min)	CH	10	70.9 ± 9.3	69.0 ± 8.7	70.7 ± 10.0	139.3 ± 7.6	147.7 ± 10.4	n.s.
	PL		69.3 ± 6.5	66.6 ± 5.3	69.2 ± 6.7	138.1 ± 8.3	149.0 ± 11.5	
Mean arterial blood pressure (mmHg)	CH	7	79.6 ± 4.2	78.0 ± 6.8	76.3 ± 6.8	86.5 ± 5.9	-	n.s.
	PL		78.3 ± 5.0	76.1 ± 4.6	76.5 ± 7.7	85.3 ± 7.2		
Body core temperature (°C)	CH	9	36.73 ± 0.76	36.50 ± 0.77	36.48 ± 0.68	36.72 ± 0.53	37.03 ± 0.40	n.s.
	PL		36.48 ± 0.76	36.34 ± 0.73	36.33 ± 0.71	36.55 ± 0.61	36.87 ± 0.60	
Mean skin temperature (°C)	CH	8	31.52 ± 0.71	31.81 ± 0.88	32.06 ± 0.65	34.20 ± 0.91	34.67 ± 0.84	*P* = 0.035
	PL		31.82 ± 0.98	31.98 ± 0.84	32.03 ± 0.82	34.00 ± 0.80	34.45 ± 0.58	
Mean body temperature (°C)	CH	7	35.58 ± 0.58	35.45 ± 0.56	35.49 ± 0.50	36.15 ± 0.45	36.52 ± 0.35	n.s.
	PL		35.50 ± 0.75	35.45 ± 0.72	35.45 ± 0.69	36.02 ± 0.56	36.37 ± 0.51	
Skin blood flow (AU)	CH	10	0.084 ± 0.028	0.079 ± 0.015	0.082 ± 0.021	0.482 ± 0.326	0.699 ± 0.421	n.s.
	PL		0.109 ± 0.046	0.094 ± 0.032	0.107 ± 0.034	0.491 ± 0.197	0.764 ± 0.338	
CVC (AU/mmHg)	CH	7	0.0011 ± 0.0004	0.0010 ± 0.0001	0.0010 ± 0.0003	0.0039 ± 0.0016	-	n.s.
	PL		0.0011 ± 0.0003	0.0011 ± 0.0003	0.0012 ± 0.0002	0.0039 ± 0.0013		
Plasma TAA (μM)	CH	10	2604 ± 257	-	3507 ± 344*	-	-	*P* < 0.001
	PL		2686 ± 242		2649 ± 292			
Thermal sensation (AU)	CH	10	3.2 ± 1.0	-	3.3 ± 1.4	6.6 ± 0.7	-	n.s.
	PL		3.4 ± 0.7		3.6 ± 1.3	6.5 ± 0.5		
RPE (AU)	CH	10	8.6 ± 2.2	-	9.4 ± 2.8	15.0 ± 2.3	-	n.s.
	PL		8.5 ± 2.2		9.8 ± 2.8	14.2 ± 1.5		

Values are expressed as mean ± SD. CH, casein hydrolysate; PL, placebo; CVC, cutaneous vascular conductance; TAA, total amino acids; RPE, rating of perceived exhaustion; n.s., not significant.

**Table 4:** Body core temperature and mean body temperature thresholds and slopes for sweating in the main experiment.

	Threshold (°C)				Slope (mg/cm^2^/min/°C)	
	Tco	ΔTco	Tb	ΔTb	Tco	Tb
Chest sweat rate						
CH	35.94 ± 0.44 *	0.31 ± 0.22	35.47 ± 0.44	0.69 ± 0.24	0.97 ± 0.38 *	0.79 ± 0.40
PL	35.72 ± 0.46	0.23 ± 0.22	35.26 ± 0.40	0.56 ± 0.29	0.71 ± 0.25	0.61 ± 0.29
Forearm sweat rate						
CH	35.99 ± 0.45 *	0.36 ± 0.21	35.53 ± 0.45	0.75 ± 0.26	0.79 ± 0.61	0.60 ± 0.37
PL	35.77 ± 0.40	0.29 ± 0.28	35.30 ± 0.31	0.60 ± 0.40	0.62 ± 0.32	0.59 ± 0.31

Values are expressed as mean ± SD. CH, casein hydrolysate; PL, placebo; Tco, body core temperature; Tb, mean body temperature; * *P* < 0.05.
